# Resonant Dynamics of Grounded Cognition: Explanation of Behavioral and Neuroimaging Data Using the ART Neural Network

**DOI:** 10.3389/fpsyg.2016.00139

**Published:** 2016-02-11

**Authors:** Dražen Domijan, Mia Šetić

**Affiliations:** ^1^Laboratory for Experimental Psychology, Department of Psychology, Faculty of Humanities and Social Sciences, University of RijekaRijeka, Croatia; ^2^Psychology Research Laboratory, Department of Psychology, Catholic University of CroatiaZagreb, Croatia

**Keywords:** adaptive resonance theory, functional neuroimaging, grounded cognition, neural network model, perceptual simulation

## Abstract

Research on grounded cognition suggests that the processing of a word or concept reactivates the perceptual representations that are associated with the referent object. The objective of this work is to demonstrate how behavioral and functional neuroimaging data on grounded cognition can be understood as different manifestations of the same cortical circuit designed to achieve stable category learning, as proposed by the adaptive resonance theory (ART). We showed that the ART neural network provides a mechanistic explanation of why reaction times in behavioral studies depend on the expectation or attentional priming created by the word meaning (Richter and Zwaan, 2009). A mismatch between top-down expectation and bottom-up sensory data activates an orienting subsystem that slows execution of the current task. Furthermore, we simulated the data from functional neuroimaging studies of color knowledge retrieval that showed anterior shift (Chao and Martin, 1999; Thompson-Schill, 2003) and an overlap effect (Simmons et al., 2007; Hsu et al., 2011) in the left fusiform gyrus. We explain the anterior effect as a result of the partial activation of different components of the same ART circuit in the condition of passive viewing. Conversely, a demanding perceptual task requires activation of the whole ART circuit. This condition is reflected in the fMRI image as an overlap between cortical activation during perceptual and conceptual processing. We conclude that the ART neural network is able to explain how the brain grounds symbols in perception via perceptual simulation.

## Introduction

The classical approach to knowledge representation assumes that a cognitive system contains symbols that refer to an aspect of the external world. An important property of symbols is that they are amodal or detached from specific sensory or motor experiences produced by the referent objects. Another property is that symbols are arbitrarily related to their referents, that is, any symbol can represent anything in the world; however, the specific meaning that will be attached to the particular symbol is a matter of convention (Markman and Dietrich, 2000). These properties of symbolic representation allow great flexibility in modeling cognitive processing because they reduce the computational burden and allow the focus to be on the abstract relations between symbols (Fodor and Pylyshyn, 1988). However, purely symbolic representations suffer from the symbol grounding problem; that is, they are unable to tie the meaning of the symbol to its referent object (Harnad, 1990; Glenberg and Robertson, 2000).

### Grounded cognition

An alternative approach to knowledge representation known as grounded or embodied cognition suggests that abstract symbols are not detached from the perceptual (or more generally experiential) traces related to the objects they represent (Glenberg et al., 2013). According to the theory of perceptual symbol systems (PSS), a symbolic representation of the concept retains certain features of the content of the perceptual experience produced by the object to which the concept refers. The central assumption of the PSS theory is that concept retrieval reactivates the memory traces of past perceptual experiences associated with denoting an object (Barsalou, 1999, 2003). Such a reactivation is called a perceptual simulation, and it solves the symbol grounding problem by using a bidirectional flow of information between perceptual and conceptual representations. Perceptual simulation is closely related to mental imagery because it produces quasi-perceptual experiences. However, an important difference is that mental imagery is an effortful, conscious activity, whereas perceptual simulation is automatically activated whenever a concept is called to mind (Barsalou et al., 2003). Similar ideas have been expressed by Glenberg and Robertson (1999) in an indexical hypothesis and by Zwaan (2004) in the model of the immersed experiencer in sentence comprehension.

Many behavioral studies have provided support for the claims of the theories of grounded cognition (reviewed in Zwaan, 2004; Pecher and Zwaan, 2005; Gibbs, 2006; Barsalou, 2008). For instance, in one of the earliest experiments, Stanfield and Zwaan (2001) used sentences such as

“He hammered a nail to the wall”

or

“He hammered the nail into the floor.”

The first sentence implies that the nail is oriented horizontally. Conversely, the second sentence implies that the nail is oriented vertically. When participants read these sentences, they invoke different mental images of the orientation of the nail. If These sentences are followed by a picture of the nail, the reaction time to the image will be shorter in the match condition where the orientation of the nail in the image has the same orientation, as implied by the sentence relative to the mismatch condition, where the orientation of the nail in the image is different from the orientation implied by the sentence (Stanfield and Zwaan, 2001). A similar effect has been found for the implied shape of an object (Zwaan et al., 2002). Later studies revealed interactions between word or sentence comprehension with visual motion (Kaschak et al., 2005; Meteyard et al., 2007), color (Connell, 2007; Richter and Zwaan, 2009), or spatial position (Šetić and Domijan, 2007; Estes et al., 2008).

Perceptual simulation is not solely restricted to perceptual attributes; it extends to emotions and motor planning. For instance, Meier et al. (2004) showed that words for positive and negative emotional states are differentially processed depending on the color in which they are presented. Positive words are processed faster when they are presented in a white color relative to their presentation in black color. Conversely, negative words are processed faster when they are presented in black. A similar effect was found for spatial position because positive words were processed faster in the upper visual field, and negative words were processed faster in the lower visual field (Meier and Robinson, 2004). Similarly, comprehending sentences influences motor control, as exemplified by the action-sentence compatibility effect. When the sentence implied movement toward the observer (“Open the drawer.”), participants were faster at executing the pull movement with their hand. Conversely, if the sentence implied movement away from the observer (“Close the drawer”), participants were faster at executing a push movement (Glenberg and Kaschak, 2002).

Functional neuroimaging studies have provided partial support for perceptual simulation (Martin, 2007, 2009). Chao and Martin (1999) showed that color perception activated lingual and fusiform gyri of the occipital lobe. Interestingly, color naming activated portions of the fusiform gyrus that are close to but anterior to the site activated by color perception. However, there was no direct overlap in cortical activation during color perception and color knowledge retrieval, as the theory of PSS would predict. Later studies in other modalities also found that conceptual processing produces brain activation that is located close to but usually anterior to the areas activated during perception. This general trend observed in many neuroimaging studies of conceptual processing was labeled anterior shift (Thompson-Schill, 2003; Rugg and Thompson-Schill, 2013). Simmons et al. (2007) argued that previous studies used passive viewing, which does not activate the whole network of areas dedicated to perception. Instead, Simmons et al. (2007) employed demanding perceptual task involving luminance judgments and found overlap in cortical activation of the left fusiform gyrus during conceptual and perceptual color processing. Hsu et al. (2011) found similar overlap in cortical activation in the left fusiform gyrus and, to a lesser extent, in the left lingual gyrus. Additionally, they found that the degree of overlap between perception and conceptual processing depended on how detailed the retrieval of color knowledge is.

### Goal of the current work

An important critique of theories of grounded cognition, such as perceptual symbol systems, is that they lack formal specification (Barsalou, 2008). In other words, it is not clear what neurocomputational mechanisms are capable of performing perceptual simulation during conceptual processing. In this study, we suggest that adaptive resonance theory is an appropriate computational framework for understanding the interaction between perception and conceptual comprehension (Grossberg, 1980, 2012; Carpenter and Grossberg, 2003). The ART is firmly based on biophysically plausible neural mechanisms such as lateral inhibition, gain control and associative (Hebbian) learning. Therefore, ART offers a firm starting point to discuss the neural processing underlying grounded cognition. Preliminary results using real-time implementation of the ART circuit with simulations of the data regarding the interaction between motion perception and motion words were presented in Domijan and Šetić (2009).

In this work, we will demonstrate how the adaptive resonance theory can explain behavioral (Richter and Zwaan, 2009) and fMRI data (Thompson-Schill, 2003; Simmons et al., 2007; Hsu et al., 2011) regarding the interaction between color perception and color words and how it deepens our understanding of the neural basis of conceptual processing. We choose to model the data of Richter and Zwaan (2009) because it is a rare example of a study that includes control or neutral condition. Thus, it was possible to distinguish between two possible behavioral consequences of grounding: facilitation or faster response in the match condition (when the simulation content matches the content of perception) and interference or slower response in the mismatch condition (when the simulation content is mismatched with the content of perception). Additionally, several fMRI studies employed color as stimuli (Simmons et al., 2007; Hsu et al., 2011), providing an opportunity to unify the behavioral and neural level of analysis. In their experiments, Richter and Zwaan (2009) requested that participants make color discrimination judgments between two sequentially presented colored squares. After the presentation of the first (reference) square, but before the presentation of the second (target), participants saw a word that either denoted color or did not. The color word either denoted the color used in the color discrimination task (match condition) or did not (mismatch condition). The color words used in the study were red, green, blue, yellow, cyan and magenta. In the neutral condition, a word did not refer to any color. The neutral words were raw, great, best, yeasty, cozy, and marital. Richter and Zwaan (2009) found that reading of the color word influenced the speed of the subsequent color discrimination. In particular, participants were slower to respond in the mismatch condition relative to the match and neutral conditions. The similar effect was observed for the same and for different responses. In this study, we will demonstrate how this interference effect arises as a consequence of the dynamics of the ART neural network.

## Model description

### Design principles of the ART

The ART was designed to solve the problem of the stability of learning in a non-stationary environment (Grossberg, 1980, 2012; Carpenter and Grossberg, 1987, 2003). Many neural network algorithms are able to detect and represent statistical regularities in the input patterns. However, when input statistics are altered (as often occurs in real-life situations), old codes are quickly erased, despite the fact that they may continue be predictive and useful. This occurrence is known as catastrophic forgetting, which significantly reduces the capability of many classes of neural networks to serve as a model of human memory and concept learning (French, 1999). According to Grossberg (1980), the solution to the problem of catastrophic forgetting is to compare perceptual (bottom-up) data with learned (top-down) expectations.

In the ART, the stability of conceptual learning is achieved by division of labor between two processing subsystems: attentional and orienting subsystems. An attentional subsystem is responsible for storing activity patterns into long-term memory. When perceptual (bottom-up) and conceptual (top-down) signals are sufficiently similar, they generate resonance in the attentional subsystem, which supports memorization of current network activity. Conversely, a mismatch between perceptual and conceptual signals triggers the activation of the orienting subsystem, which sends a global reset signal to the attentional subsystem. The reset signal temporarily disables the currently active concept node and forces the attentional subsystem to search for a new node. If there is no category node that matches the input, a new concept node is dedicated to learning the current input pattern. In other words, activation of the orienting subsystem indicates that the network encounters a new input pattern. An orienting subsystem operates as a novelty detector, and it prevents the recoding of old memories when confronting new input. Thus, the orienting subsystem ensures the stability of old memories and simultaneously enables the acquisition of new ones.

Top-down expectations could be read-out from long-term memory in response to the presentation of input, or they could be initiated internally to produce attentional priming in a perceptual representation (Carpenter and Grossberg, 2003). Here, we suggest that the same top-down pathway that is needed to stabilize learning and that is responsible for attentional priming also supports perceptual simulation during conceptual processing. In other words, perceptual simulation arises from the same neural mechanisms that prevent catastrophic forgetting, that is, they prevent interference between previously established memory traces and new patterns.

### Real-time implementation of the ART neural network

**Figure 1:**
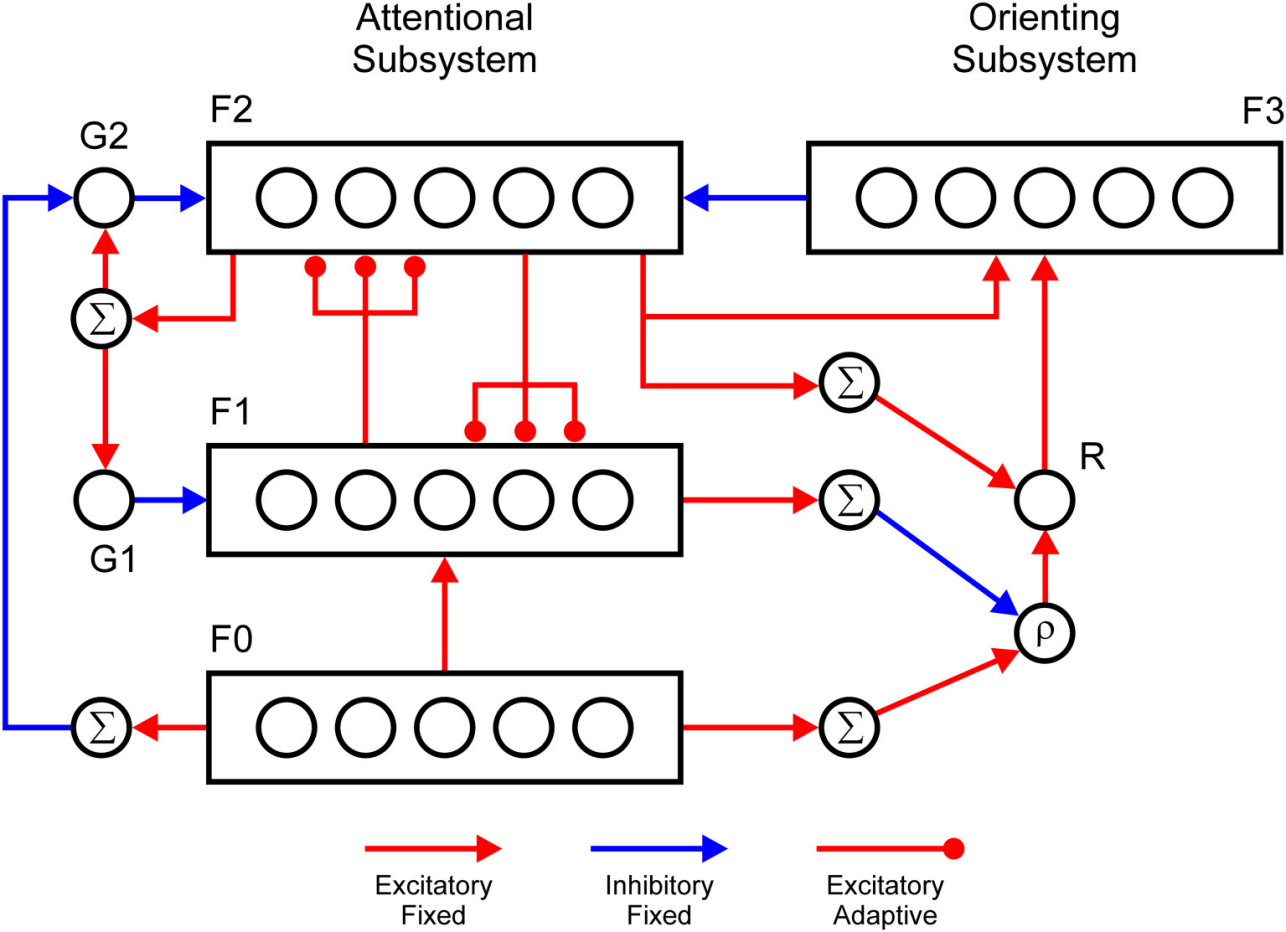
**Adaptive resonance theory (ART) circuit with two processing streams: attentional and orienting subsystems**. The attentional subsystem consists of three layers, denoted as F_0_, F_1_, and F_2_. The F_0_ layer registers the input pattern, and contrast enhances it via lateral inhibition. The F_1_ layer combines feedforward activity from F_0_ and feedback projections from F_2_ to compute the intersection between bottom-up input and top-down expectations. The F_2_ layer also employs lateral inhibition in order to choose the category node that best matches the input pattern. The gain control unit G_1_ inhibits the F_1_ layer and unit G_2_ inhibits the F_2_ layer to prevent their activation during perceptual simulation. The G_1_ and G_2_ units are excited by the activity of the F_2_ layer. Furthermore, the G_2_ unit is inhibited by the activity of the F_0_ layer. In the orienting subsystem, the R node computes the mismatch between the total amount of activity in F_0_ and F_1_. It also receives recurrent projection from the F_2_ layer. The R node sends excitatory signals to the F_3_ layer, which inhibits F_2_. The F_3_ node is activated when it simultaneously receives excitation from the F_2_ layer and the R node. Thus, the F_3_ layer specifically inhibits only the currently active node in F_2_ and ensures that it will not be activated again during the course of a trial. The R node is activated when the total amount of activity in F_0_ exceeds that in F_1_ by a threshold specified by the vigilance parameter ρ. Nodes labeled with Σ compute the sum over the activity of whole layer. Red (blue) lines denote excitatory (inhibitory) connections. Arrows indicate fixed connections, whereas disks denote adaptive connections.

Specific implementation of the ART design principles used in the current study is shown in Figure 1. A mathematical description of the model is provided in the Supplementary Material. As noted above, in the ART circuit, category learning is achieved via interaction between two complementary processing streams: an attentional and an orienting subsystem. The attentional subsystem consists of three layers of nodes, which are labeled F_0_, F_1_, and F_2_, and two gain control units, G_1_ and G_2_ (Carpenter and Grossberg, 1987, 2003). F_0_ is an input layer that registers the pattern of sensory stimulation. Additionally, it suppresses the input noise via lateral inhibition and enhances the representation of the target features via self-excitation. F_1_ reads-out the activity pattern from F_0_ and combines it with the top-down expectations arriving from the F_2_ layer. In the absence of top-down signals, the F_1_ layer can generate supra-threshold activity because the gain control unit G_1_ is not active. The activation further flows from F_1_ to F_2_ and passes through a filter of adaptive weights. Like F_0_, the F_2_ layer employs lateral inhibition in order to implement choice or winner-takes-all behavior that represents the category or the concept that best matches the sensory input. When the F_2_ layer makes a choice, the winning F_2_ node sends top-down signals to F_1_ and to the G_1_ unit, which further inhibits the F1 layer. In this case, the F_1_ layer computes logical AND between its two sources of input; that is, it attains a supra-threshold level of activity solely if it simultaneously receives excitatory input from both F_0_ and F_2_. Consequently, if the total activity in the F_1_ layer is similar to that in F_0,_ it signals that the chosen F_2_ node is a suitable match to the sensory pattern registered at F_0_. Conversely, if the chosen F_2_ node does not represent the sensory pattern well, the total activity in F_1_ will be much smaller relative to F_0_. Carpenter and Grossberg (1987) discussed four different means by which F_1_ could perform this matching. We choose the simplest one, which requires the minimum amount of neural circuitry. The second gain control unit, G_2_, inhibits the F_2_ layer and prevents its full activation in the absence of sensory input, similar to how the G_1_ unit does this for the F_1_ layer. Furthermore, the G_2_ unit is inhibited by the activation of the F_0_ layer, which further disinhibits the F_2_ layer. Thus, the gain control mechanisms, G_1_ and G_2_, enable a distinction to be made between sensory stimulation and internal activation.

The orienting subsystem consists of the reset node, R, and a layer, F_3,_ of nodes that deliver specific inhibition to the F_2_ layer. The R node computes the ratio between the total activation in F_0_ and F_1_. The node produces a reset signal when this ratio is smaller than a threshold denoted as a vigilance parameter ρ. The vigilance parameter controls the precision of the category coding, that is, how similar patterns should be to prevent the activation of the orienting subsystem. When activated, the R node delivers non-specific excitation to the F_3_ layer. The F_3_ layer has the same dimensionality as F_2_, and they are mutually connected in a one-to-one manner. Each F_2_ node sends excitation to its corresponding F_3_ node and receives inhibition from it. The F_3_ node becomes active solely if it simultaneously receives two sources of excitation. One source is the activity of the F_2_ node, and a second source is activity from the R node. When activated, the F_3_ node inhibits its twin F_2_ node and initiates a search for another F_2_ node that will provide a better match to the sensory pattern. Thus, the orienting subsystem enables learning of a new category by inhibiting F_2_ nodes that are already committed to encoding familiar categories.

One problem that arises during the real-time operation of the orienting subsystem is that F_0_ is activated before F_1_ on the first wave of the signal flow through the attentional subsystem. Therefore, there is a short temporal window during which there is a mismatch between the activity of F_0_ and F_1_ that is not a consequence of the mismatch between sensory and top-down signals in F_1_ because F_1_ is not activated yet by F_0_. To prevent premature activation of the orienting subsystem, we introduced a recurrent projection from the F_2_ layer to the R node. This idea was first proposed by Ryan and Winter (1987). Additionally, the R node receives a tonic inhibition, which prevents its activation by a mismatch signal alone. Consequently, the R node will become active solely when it simultaneously receives a signal from F_2_ and a mismatch signal. Thus, a reset signal could be delivered to the attentional subsystem solely at moments when the F_2_ layer is already active.

### Interaction between the ART modules

**Figure 2:**
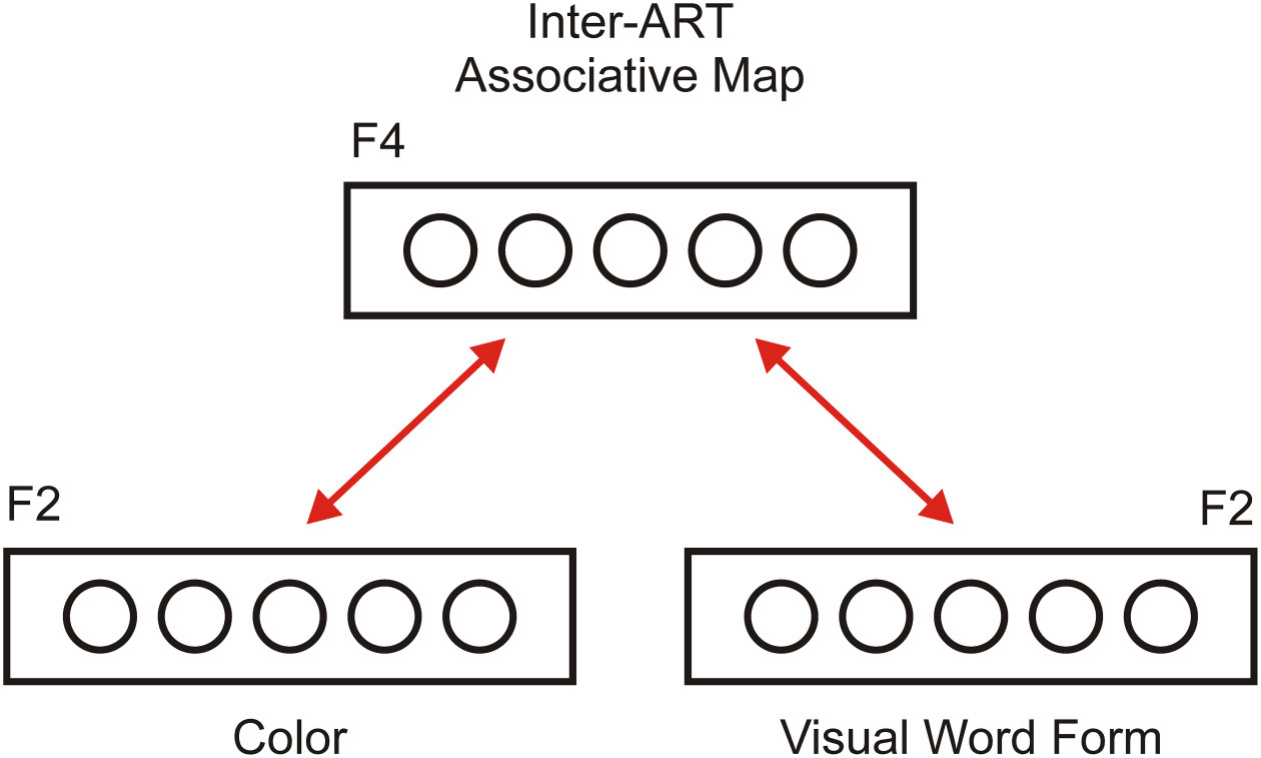
**Inter-ART associative map (or F_**4**_ layer) as a link between two ART modules**. One ART module is dedicated to color perception and categorization (Color module), and the second module is dedicated to written word recognition (Visual Word Form module). Each ART module has bidirectional excitatory connections with the associative map. Therefore, activity in one ART module can produce perceptual simulation in other module (supra-threshold activation of the F_2_ layer and sub-threshold activation of the F_1_ layer). The inter-ART associative map provides a solution to the symbol grounding problem because it connects a symbolic representation of color words with a perceptual representation of the colors denoting them.

One ART circuit is capable of learning many categories in a stable manner; however, it cannot attach symbolic labels to learned categories. To solve this problem, Carpenter et al. (1991) proposed an ARTMAP architecture that describes how two ART modules arising from different input modalities can interact with each other via an inter-ART associate map. We adopted the same architecture to explain the interaction between color perception and color words, as shown in Figure 2. In the model, the color module is responsible for color perception and discrimination. Resonance in this module indicates that the participant has successfully discriminated a particular hue of color as familiar. The visual word form (VWF) module is responsible for visual word recognition. In this module, resonance develops between letter shapes in the F_1_ layer and the appropriate recognition node in its F_2_ layer. Therefore, resonance in the VWF module indicates that the participant has successfully recognized a presented pattern of lines as a familiar word.

Importantly, two modules are bi-directionally connected via an inter-ART associative map, denoted as the F_4_ layer, which serves as a hub that directs communication between different ART modules. Thus, neural activity in one module could spread toward other modules if they share the same concept. For instance, the word RED in the VWF module could activate the F_2_ node, which represents red in the color module, although it is not directly provided in the input. Thus, color processing in the color module could activate the F_2_ layer in the VWF module, thus creating an expectation regarding the word that could be registered. We argue that this spreading activation between modules constitutes a neural basis for perceptual simulation, as described by the theory of perceptual symbol systems (Barsalou, 1999). In other words, an inter-ART associative map solves the symbol grounding problem by binding activity from an abstract representation of color words with the perceptual representation of colors. Of course, it is possible to extend this architecture with additional ART modules encoding other modalities, such as auditory, tactile, gustatory, and modules for emotions and motor actions. Interestingly, the structure described in Figure 2 is similar to the model of semantic memory proposed by Patterson et al. (2007).

### Input representation

**Figure 3:**
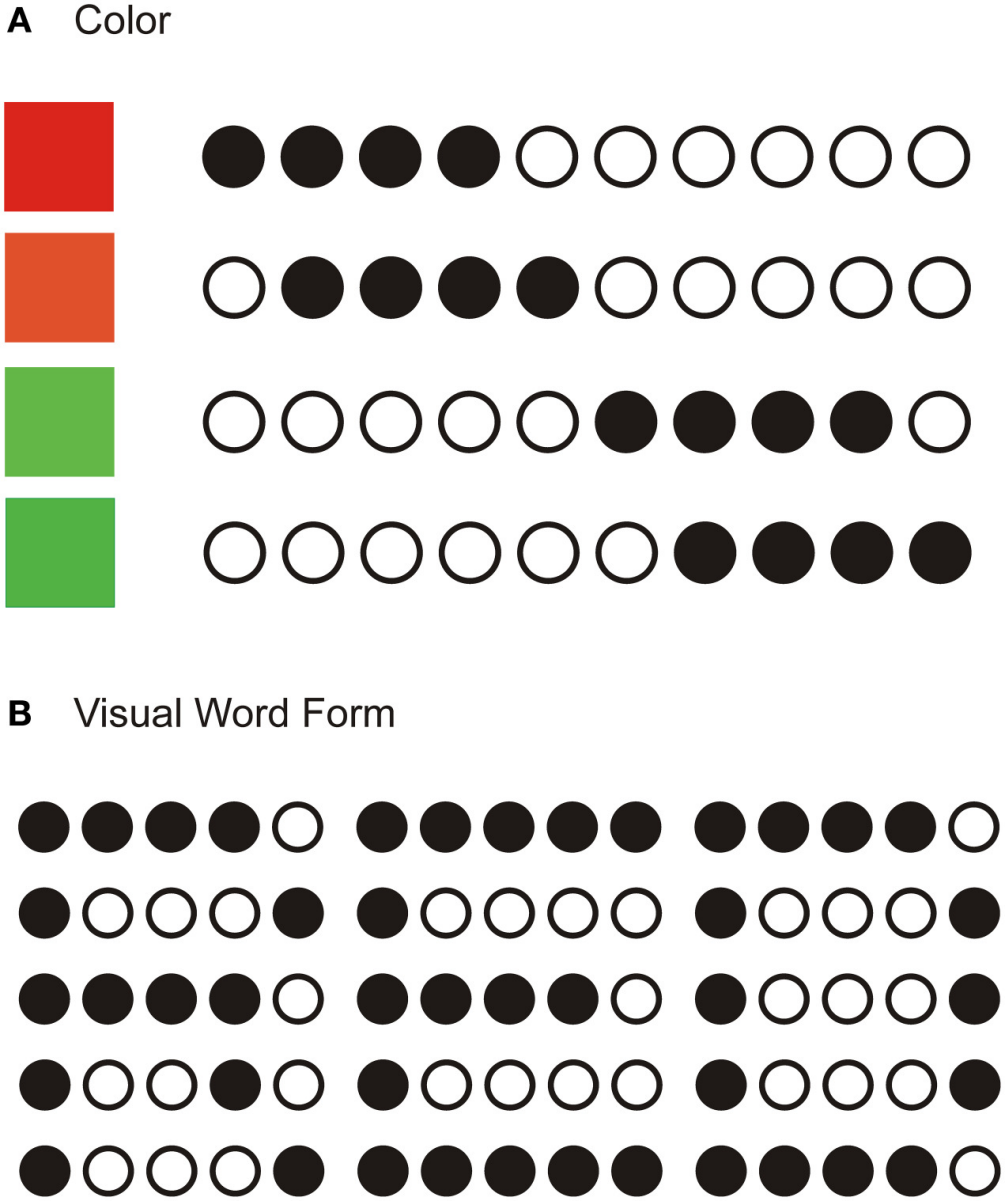
**Structure of the F_**0**_ and F_**1**_ layers in the color module (A) and in the visual word form module (B)**. In **(A)**, activity in a one-dimensional array of nodes represents different hues of color. Similar colors produce overlapping patterns, whereas dissimilar colors produce non-overlapping patterns of activation. In **(B)**, a two-dimensional array of nodes represents written words. Black disks denote active nodes, whereas empty circles denote inactive nodes.

Figure 3 illustrates input representations for colors and words in their respective ART modules. Colors are represented as distributed activations of sets of nodes in the F_0_ and F_1_ layers. Similar hues are represented by overlapping patterns of activation, and the degree of similarity is reflected in the degree of overlap in activation (Figure 3A). When resonance develops in the ART color module, it means that the participant recognized the presented color. How precise the color discrimination is depends on the vigilance parameter. When the vigilance parameter is set to a high value, the network is able to discriminate among highly overlapping patterns. In the VWF module, words are represented as template-like two-dimensional patterns (Figure 3B).

### Behavioral output

**Figure 4:**
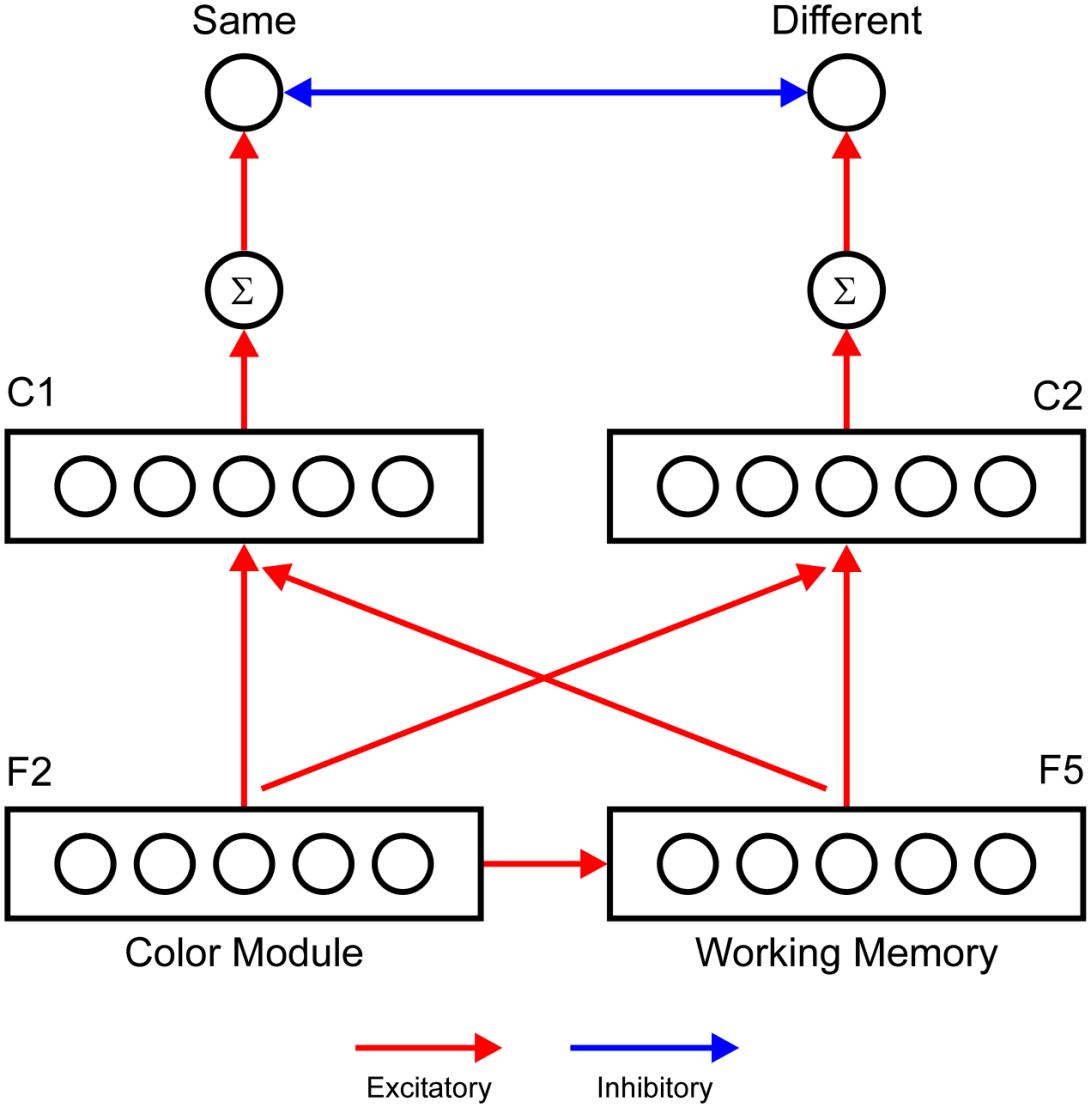
**A neural network designed to generate a behavioral response in the perceptual discrimination task based on the match of content in F_**2**_ and in F_**5**_ or the working memory layer**. Two comparison layers (C_1_ and C_2_) receive topographically organized input from the F_2_ and F_5_ layers. The C_1_ layer computes logical AND between F_2_ and F_5_ outputs. Therefore, the node in C_1_ becomes active only when both F_2_ and F_5_ nodes at the same network location are active. Conversely, the C_2_ layer computes logical OR. The node in C_2_ becomes active, although one of the nodes (either F_2_ or F_5_) is active. The response units (Same and Different) are modeled as leaky integrator units that accumulate evidence for the particular response alternative. The Same unit will be activated if one of the C_1_ nodes is active, providing evidence that the F_5_ activity matches that in the F_2_ layer. The Different unit has an elevated threshold because it should be activated only if two separate C_2_ nodes are simultaneously active, indicating that the content of F_5_ is mismatched with the current F_2_ activity.

To generate a model output that is comparable to human performance and to simulate the data of Richter and Zwaan (2009), we introduce two extensions with respect to the architecture of the ART circuit depicted in Figure 4. First, we introduce a working memory layer, denoted as F_5_, that holds information about previous F_2_ activity over the course of a trial. We assume that there is a one-to-one mapping from the F_2_ layer to the F_5_ layer. Therefore, each F_2_ node has its own F_5_ node. The F_5_ layer tracks the color of the stimulus that was presented first (reference stimulus) in the color discrimination task, which involves the sequential presentation of stimuli. Conversely, the F_2_ activity encodes the color of the stimulus presented second (target stimulus).

Second, we introduce a response network with two output units that simulate decision making and response preparation. Output units are designed to mimic the gradual accumulation of evidence for particular response alternatives, as described by the leaky competitive accumulator model (Usher and McClelland, 2001). The network is considered to have made a response when one of the output units exceeds the threshold for activation that triggers motor execution. Output units allow direct comparison with behavioral data because they enable simulation of reaction time data. In the color discrimination task, there are two possible responses: same or different, depending on whether the two stimuli matched in color or not, respectively. Therefore, we employed two response units: the Same and Different accumulator units. The Same unit receives excitatory input from the comparison layer, C_1_. Nodes in the C_1_ layer compute logical AND between inputs they receive from the F_5_ and F_2_ layers. Each C_1_ node receives input from one F_5_ node and from one F_2_ node, which are positioned at the same location within the F_5_ and F_2_ layers. The computation of function AND is achieved by the threshold, which is set to an elevated value. An elevated threshold ensures that the C_1_ node will become active only if it simultaneously receives excitation from the F_2_ and F_5_ nodes. Effectively, the C_1_ layer detects juxtaposition of the neural activity in the F_2_ and F_5_ layers. This will occur when the colors of two stimuli are the same. Conversely, the Different unit receives input from the C_2_ layer, which computes logical OR between inputs from the F_2_ and F_5_ layers. The threshold for activation of the Different unit is also set to an elevated level. Thus, the Different unit will become active only if it receives simultaneous excitation from two distinct C_2_ nodes. This will occur when the F_5_ and F_2_ layers are active at different network locations, indicating that the colors of the referent and target stimuli are different.

## Results

### Simulation of behavioral data of Richter and Zwaan (2009)

Several processes occur before the start of the simulation, and these are explained first. At the beginning of each trial, a reference color is presented that activates the ART color module. When resonance occurs in the color module, activity from the F_2_ layer is loaded into the F_5_ (working memory) layer, which retains it for future comparison with the color of the target square. Subsequently, when a participant reads a word, an ART module for visual word form recognition is activated. When resonance in the VWF module is established, that is, when the VWF module recognizes the presented shape as a familiar word, the currently active F_2_ node sends excitation to the corresponding node in the associative map. Furthermore, the activated node in the associative map sends excitation to the F_2_ layer of the color ART module. Thus, the associative map sends top-down expectation to the color module consistent with the concept activated by the color word. In other words, perceptual simulation of the color is generated in the color module related to the meaning of the color word. This situation is illustrated in **Figure 6** for three experimental conditions: (A) matching color word—RED; (B) mismatching color word—GREEN; and (C) neutral word—BEST. It is important to note that the mapping between the F_2_ layer in the color module and the associative map is not one-to-one because different hues of the same color could be perceptually distinguished, although we cannot name it differently. In other words, the associative map is able to group different hues of the same color under the same color label. Therefore, the same node in the associative map activates several nodes in the F_2_ layer of the ART color module.

**Figure 5:**
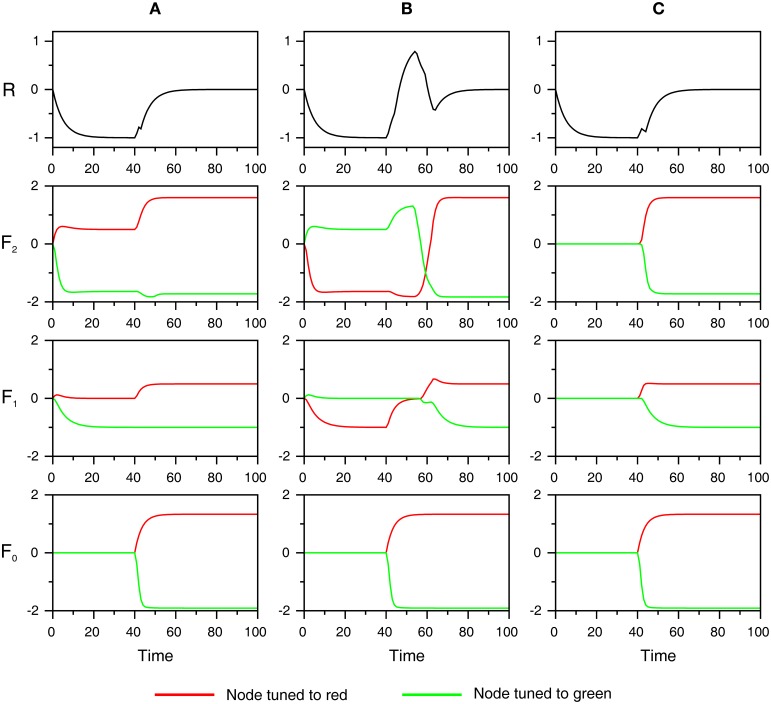
**The time course of neural activity of the different components of the ART color module is shown for three experimental conditions used in behavioral study of Richter and Zwaan (2009): matching color word (A), mismatching color word (B), and neutral word (C)**. Red and green lines show the activity of the node tuned to red and green, respectively. At the beginning of the simulation, color words create expectations by activating corresponding nodes in the F_2_ layer. In **(A)**, the word RED activates the node for red, will be presented subsequently. In **(B)**, the word GREEN activates the node for green, which will not be shown later. In **(C)**, there is no top-down expectation because a neutral word such as BEST does not have access to the color module. At time point *t* = 40, input consisting of a red square is registered at F_0_. In **(A,C)**, there is no mismatch between the input pattern and top-down expectation; thus, there is no supra-threshold activation of the R node. Conversely, in **(B)**, the input mismatches with the top-down signals, thus activating the R node, which inhibits the trace of erroneous expectation from the F_2_ layer.

Figure 5 shows the time course of activation of the components of the ART color module. At the beginning of simulation, expectations arising from the associative map are read-out at the F_2_ layer. Due to the tonic inhibition from the G_2_ node, activity in the F_2_ layer is weak but supra-threshold so that the F_2_ layer can excite the F1 layer. Conversely, activity in the F_1_ layer is sub-threshold because the excitation from the F_2_ layer is canceled by an equal amount of inhibition from the G_1_ node. Here, it is assumed that the G_1_ inhibition is always strong enough to keep the F_1_ layer below the threshold. Therefore, its weakening may lead to resonance, although no stimulus is present. In other words, activation of the F_1_ layer without accompanying sensory stimulation from F_0_ leads to hallucinations.

At time point *t*_1_ = 40, a red square is presented that activates the nodes in the F_0_ layer tuned to the red color. In the match condition (Figure 5A), the top-down expectation from the F_2_ layer is confirmed by the sensory input from the F_0_ layer. In this case, the F_1_ layer is sufficiently active to prevent (inhibit) activation of the R node. The R node exhibits sub-threshold activation arising from the F_2_ layer, but this activation never coincides with the mismatch signal. Consequently, the F_2_ node tuned to red quickly reaches its maximal level of activity (or reached resonant state) without any interruption from the orienting subsystem. It only receives disinhibition from the G_2_ unit, which is inhibited by the activity from F_0_ when it encodes the input.

In the mismatch condition (Figure 5B), where the F_2_ layer expects the green color, but red is registered at F_0_, the R node attains a supra-threshold level of activity and inhibits the traces of erroneous expectation in the F_2_ layer. The R node becomes active because the mismatch signal is combined with the top-down signal from F_2_. The mismatch signal arises because the F_1_ layer simultaneously receives two sources of input, but it produces no output because these input sources do not match each other. A consequence of the activation of the orienting subsystem is that the F_2_ node tuned to green is inhibited, which allows the F_2_ node tuned to red to become active. However, this node reaches its maximal level of activity at a later time point relative to that in the match condition. Finally, if there is no expectation created by the word as it occurs in the neutral condition (Figure 5C), there is also no supra-threshold activation of the R node as in the match condition. Therefore, the F_2_ node tuned to red reaches resonant state at a point of time that is much earlier relative to the mismatch condition and, to some extent, later relative to the match condition.

**Figure 6:**
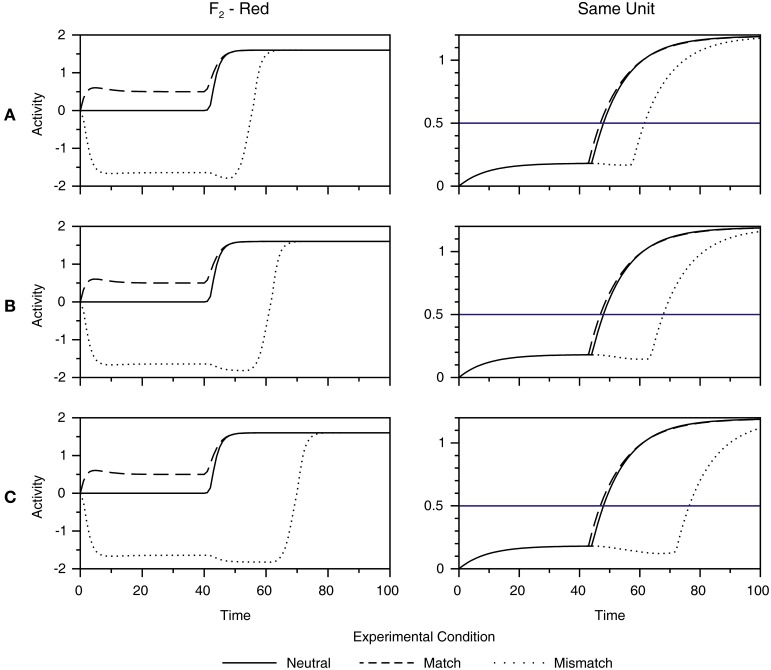
**Simulation of reaction times in a behavioral experiment of Richter and Zwaan (2009)**. Motor output is obtained by transformation of the activity of the F_2_ node tuned to red (left column) into the activity of the Same unit in the response network (right column). Furthermore, we examined how the dynamics of the F_2_ layer and the response network are affected by systematic variation of the speed of the activation of the R node in the orienting subsystem, ranging from fast **(A)**, to medium **(B)** to slow **(C)**. Each graph compares activity in three conditions: match (dashed line), mismatch (dotted line), and neutral (full line). A horizontal blue line in the right column depicts The threshold for making the same response. As observed, facilitation in the match condition remains weak, irrespective of the speed of the R node. On the other hand, interference in the mismatch condition becomes stronger as the R node becomes slower, suggesting that the activation of the orienting subsystem in the mismatch condition is responsible for creating the behavioral effect of perceptual simulation.

Figure 6 shows how the dynamics of the F_2_ layer is transformed into the behavioral response (same or different) in the response network. The left column of Figure 6 (labeled F_2_—Red) redraws the time course of the activation of the F_2_ node, tuned to red, to directly compare responses in three experimental conditions (i.e., match, mismatch, and control). Furthermore, it shows how systematic variation of the speed of activation of the R node in the orienting subsystem from fast (Figure 6A), to medium (Figure 6B), to slow (Figure 6C) influences the magnitude of the facilitation and interference effect. In particular, it shows that the amount of facilitation in the match condition remains weak under changes of the time constant of the R node. On the other hand, interference in the mismatch condition becomes stronger as the R node becomes slower.

The right column of Figure 6 (labeled as Same Unit) shows the dynamics of the accumulation of evidence of the Same unit until it reaches the threshold for response depicted by the horizontal blue line. However, it should be noted that the same explanation holds for the Different unit as well. In the mismatch condition, the delay in the activation of the appropriate F_2_ node is reflected in the similar delay in the activation of the Same unit. Conversely, facilitation in the match condition remains small. When we transformed simulated time units into a millisecond scale via Equation (22), given in the Supplementary Material, we found a negligible facilitation of just 3 ms in the match condition under all three settings of the speed of the R node. On the other hand, interferences of 42, 60, and 87 ms were found in the mismatch condition with fast, medium and slow dynamics of the R node, respectively. In experiment 1, Richter and Zwaan (2009) also observed a weak facilitation of approximately 15 ms, but it was statistically unreliable in comparison with the strong interference of 51 ms in the mismatch condition. Importantly, the weak facilitation and strong interference observed in the model's output is not a byproduct of a particular choice of parameter values used to generate behavioral response in Equation (22). For instance, the threshold for motor response can be set anywhere between 0.2 and 1 without affecting the asymmetry between the magnitude of the facilitation and interference effect. To conclude, our simulation suggests that the behavioral consequence of grounding is interference in the mismatch condition, which is attributed to the activation of the reset signal in the ART circuit.

### Simulation of the anterior shift and overlap effect in functional neuroimaging

**Figure 7:**
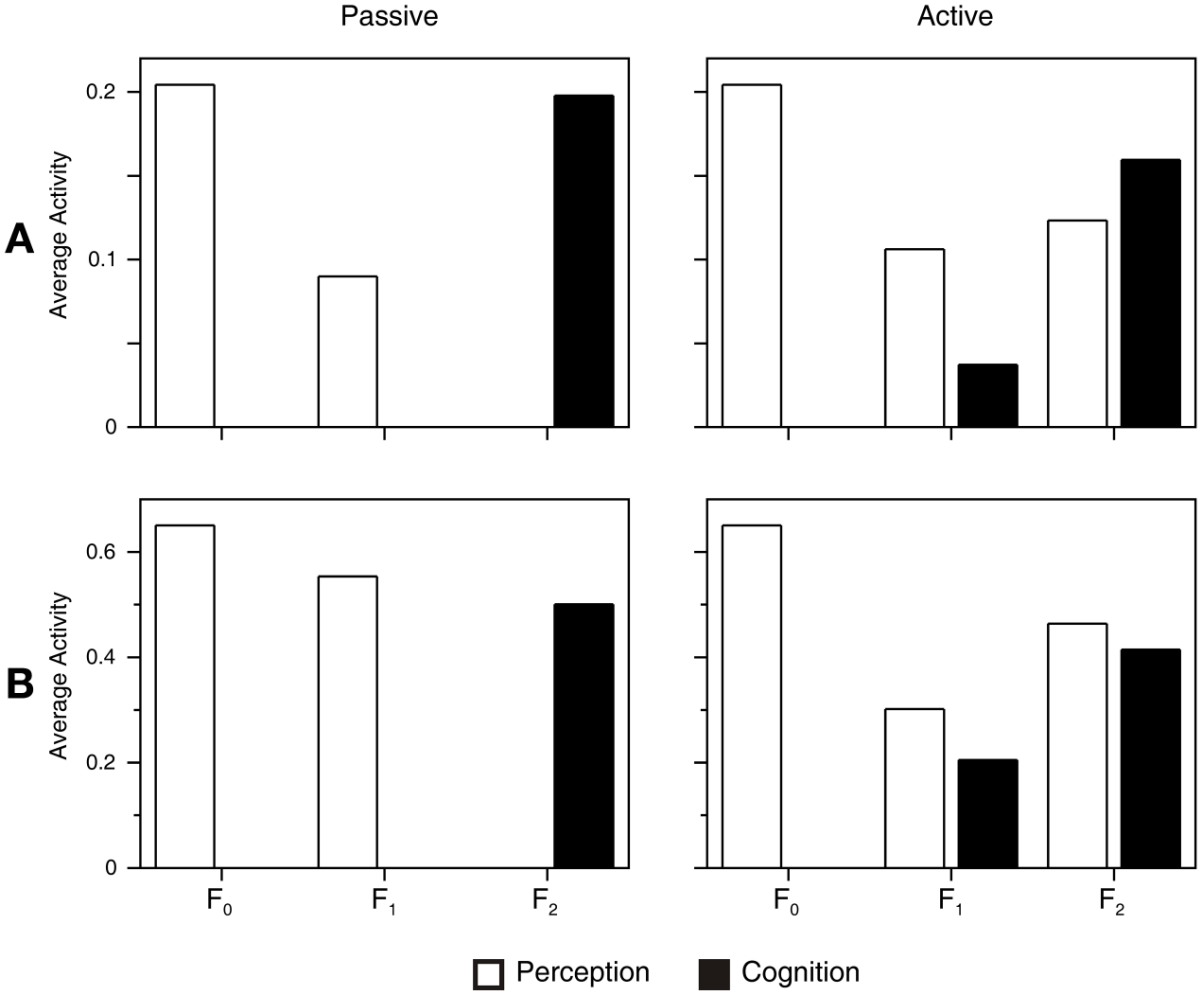
**Simulation of the hemodynamic response under different task conditions (Passive vs. Active)**. We considered the space-time average of supra-threshold activity **(A)** and the space-time average of the absolute value of membrane potential **(B)** as an index of metabolic demand and cerebral blood flow. Both measures produce similar results. Passive perception activates only F_0_ and F_1_. Conversely, passive cognition (or perceptual simulation) activates only F_2_. In a simulated metabolic response contrasting perception with cognition, this situation would appear as non-overlapping areas, thus producing the anterior effect (Chao and Martin, 1999; Thompson-Schill, 2003). When the network is engaged in a demanding perceptual task, all three layers of the attentional subsystem are activated. Conversely, active cognitive processing produces a signal in F_1_ and F_2_ layers. In the simulated metabolic response, this would appear as an overlap between perception and cognition in F_1_ and F_2_, as observed by Hsu et al. (2011) and Simmons et al. (2007).

The anterior shift observed in the left fusiform gyrus in neuroimaging studies of color knowledge retrieval (Chao and Martin, 1999; Thompson-Schill, 2003) is explained by the observation that the ART circuit is a hierarchical network composed of three processing layers, as shown in Figure 1. If this processing hierarchy is stretched on the cortical surface of the fusiform gyrus along the posterior-anterior dimension, the F_0_ layer will be positioned posterior to the F_1_ layer, and the F_1_ layer will be positioned posterior to the F_2_ layer. Figure 7A illustrates how averaged supra-threshold activity in the ART circuit generates a hemodynamic response. Here, we assumed that the passive condition produces additional inhibition in the gain control nodes, G_1_ and G_2_, as explained in the Supplementary Material. The activity of G_1_ is counted as part of the F_1_ layer and activity of G_2_ is counted as part of the F_2_ layer. Column Passive in Figure 7 shows that passive perceptual processing activates only the F_0_ and F_1_ layers because there is no need for further classification of input patterns. Therefore, the activation of F_2_ is prevented by gain control G_1_, which suppresses the output of the F_1_ layer. However, gain control node G_1_ is itself active in this condition. Therefore, activation of both the F_0_ and F_1_ layers should be observed. Conversely, passive conceptual processing will activate only the F_2_ layer because its gain control node G_2_ is active in this condition and it suppresses the activity in F_2_. When network activities during passive perception and passive cognitive tasks are contrasted with each other, the result is the anterior shift in cortical activation during cognition relative to perception. Importantly, our simulation suggests that the existence of the anterior shift should not be taken as evidence against grounding of conceptual processing (Chatterjee, 2010; Rugg and Thompson-Schill, 2013). Rather, it reveals two distinct sides of the same neural network responsible for symbol grounding.

Active perceptual processing, as it occurs, for instance, during perceptual discrimination, requires engagement of the whole processing hierarchy (column Active in Figure 7). In this case, perception activates the F_0_, F_1_, and F_2_ layers because the network must classify perceptual inputs into separate categories, and this is possible only by engagement of the F_2_ layer. During active cognitive processing, the F_1_ and F_2_ layers both show increased metabolic response. F_2_ is active because of the excitatory signals arriving from the associative map. On the other hand, F_1_ exhibits a positive signal because of the activation of its gain control node G_1_, which inhibits the F_1_ layer. When brain activity during perception and conceptual processing are contrasted against each other, we observe an overlap in cortical activation in F_1_ and F_2_. However, it should be noted that the overlap in the F_2_ layer is not perfect because the F_2_ activity during perceptual simulation is more widespread compared with focused activity during perception. As explained above, the inter-ART associative map is connected to more than one node in the F_2_ layer. For this reason, additional cognition-related F_2_ activity that is not present during perception should produce more-distributed activation in F_2_.

The above analysis rests on the assumption that the fMRI signal directly reflects neural activity, that is, the neuron's firing rate (Heeger and Ress, 2002; Nir et al., 2008). However, this may not be true. There is evidence that the fMRI signal better correlates with the local field potential, which reflects processes in the neuron's input zone rather than the neuron's firing rate (Logothetis, 2002; Logothetis and Wandell, 2004). One hypothesis is that the fMRI represents total synaptic activity because transmitter synthesis and release are energy-demanding processes (Horwitz et al., 1999; Arbib et al., 2000). Another possibility is that dendrites are responsible for generating the fMRI signal (Lauritzen, 2005; Domijan, 2011). Under these interpretations, the fMRI signal reflects processing pathways rather than neural outputs. However, Figure 7B shows that even if the absolute value of membrane potential is taken as an index of metabolic demand, the resulting pattern of cortical activation remains similar. In conclusion, irrespective of the exact source of the fMRI signal, activation of the ART circuit in response to the passive perception or cognition should produce an anterior effect (Thompson-Schill, 2003). Conversely, demanding perceptual or cognitive tasks should produce overlap in brain activity during perception and perceptual simulation (Simmons et al., 2007).

## Discussion

Our simulations show how adaptive resonance theory addresses a number of issues in the study of grounded cognition. First, properties of the ART circuit explain why grounding exists at all. In the ART, grounding is a consequence of the solution to the stability-plasticity dilemma, that is, it is a consequence of the neural mechanisms that enable stable category learning. Stability is achieved by the matching of bottom-up input and top-down expectations. We argue that the same anatomical route used to read-out top-down expectations (feedback projections from the F_2_ to F_1_ layers) is co-opted by the cognitive system to perform perceptual simulations during conceptual processing. Second, the behavioral effects of grounding arise from the activation of the orienting subsystem (reset signal) that is triggered whenever bottom-up and top-down signals mismatched. The reset signal inhibits the currently active node in the F_2_ layer and allows the new node to become active. This process requires time and consequently slows down the execution of the ongoing task.

Third, the ART explains why neural structures involved in perception and cognition should never perfectly overlap. If there were a strong overlap between perception and knowledge, the cognitive system would not be able to distinguish between perception and hallucination. This point was made by Martin (2009). For this reason, the ART network does not have feedback projections from the F_2_ to F_0_ layers or from the F_1_ to F_0_ layers. Therefore, the F_0_ layer could not be perturbed by the top-down signals. This prediction of the model is in contrast with fMRI studies showing attentional influence on the visual cortex (Kok et al., 2013; Vandenbroucke et al., 2014). However, it should be noted that electrophysiological studies have revealed that some, but not all, neurons in the visual cortex are susceptible to top-down modulations. For instance, Pooresmaeili et al. (2010) found that approximately half of the neurons in the primary visual cortex were modulated by attention but the other half were not. Several studies have found a similar division between neurons in the V4 (Luck et al., 1997; Reynolds et al., 2000). In the context of the ART circuit, neurons that are not influenced by attention are part of the F_0_ layer, which provides a veridical or reference signal that is supplied to the orienting subsystem for detecting possible match or mismatch with the top-down signals. On the other hand, neurons that are modulated by attention are part of the F_1_ layer, which serves as a matching point between bottom-up and top-down signals.

Nevertheless, even if it turns out to be true that all neurons in the visual cortex are subject to top-down modulations, the prediction of the current model is that the source of feedback signals to F_0_ should originate from a region outside the ART circuit that is dedicated to semantic knowledge. One such source of feedback signals might be fronto-parietal network (Ptak, 2012). For instance, in a multi-object scene, F_0_ would initially register features of all objects present. In this case, feature-based attention to F_0_ may increase the saliency of one object and filter out features of other objects, thus preventing the superposition catastrophe (von der Malsburg, 1999). Conversely, feedback projections from F_2_ to F_1_ solve a different computational problem, that is, the stability of learning of a selected object (Grossberg, 1980).

An important point from the simulation presented in Figure 7 is that we show how the anterior shift arises under a condition of passive perception or cognition (Thompson-Schill, 2003). In this case, perception activates the F_0_ and F_1_ layers, whereas conceptual processing activates only the F_2_ layer. When we consider both cases together, there is no overlap in the brain activation during perception and knowledge retrieval. Therefore, the anterior effect should not be used as evidence against grounding. Conversely, a partial overlap between perception and conceptual processing is observed when a more demanding perceptual or conceptual task is employed, as in the studies of Simmons et al. (2007) and Hsu et al. (2011). In this case, perception engages the whole ART network, including the F_0_, F_1_, and F_2_ layers, whereas the conceptual processing activates the F_1_ and F_2_ layers, thus creating the overlap in the fMRI image.

In contrast with other computational approaches to grounded cognition, which emphasize the beneficial effect of matching top-down expectations with sensory-motor states (Roy, 2005; Pezzulo et al., 2011, 2013; Hoffman, 2012), the present work suggests that the behavioral effect of grounding is primarily observed in the mismatch condition where expectations are not confirmed by the current sensory input. For instance, Hoffman (2012) showed how an artificial agent achieves faster visual recognition when primed by the auditory percept. Early behavioral studies (Stanfield and Zwaan, 2001; Zwaan et al., 2002) were unable to disentangle the effect of facilitation in the match condition from interference in the mismatch condition because they did not have a neutral or control condition for comparison. Richter and Zwaan (2009) solved this issue by using neutral words that do not produce perceptual simulation in the relevant color dimension. The researchers found that the grounding effect arises from the mismatch condition, where the performance in perceptual discrimination is slower compared to that in the match and neutral conditions. Conversely, the match and neutral conditions did not differ in latency. This raises the possibility that the reset signal is responsible for generating this interference effect, as shown in the simulation presented in Figure 5.

A testable prediction derived from the simulation of behavioral data is that a similar interference in the mismatch condition without corresponding facilitation in the match condition should be observed in other modalities (e.g., motion perception, spatial representation, emotion) when a neutral condition is employed together with the match and mismatch conditions. Another prediction of the model is that comprehension of conceptual combinations requires multi-modal simulation involving two or more ART modules. For instance, perceptual simulation of the concept BLACK DOG should involve attentional priming in the color module as well as in the shape module. Subsequent presentation of the picture of a brown dog or black cat should produce similar response delays because both pictures violate part of the combined expectation, thus triggering the reset signal within one of the primed ART modules. The same reset mechanism may also be involved in generating the modality switching cost in a property verification task (Pecher et al., 2003). When participants are requested to verify whether a given property belongs to the concept, they are slower when the verified property comes from a different modality relative to the property in the previous trial. An explanation of the modality switching cost would require that the inter-ART associative map have its own reset mechanism, which would enable shifting of the attentional focus between different ART modules.

With respect to the functional neuroimaging, the current model predicts that activation of the orienting subsystem in the mismatch condition along with the activation of attentional subsystem should light up several new voxels in the left fusiform gyrus in addition to those already activated by the match or neutral condition. In a similar vein, it is expected that attentional switching between modalities that occurs during property verification should light up new voxels in the anterior temporal lobe in addition to those already activated by the property verification in the same modality.

Meteyard et al. (2012) offered a useful taxonomy of theoretical viewpoints regarding the role of embodiment in cognition and its neural underpinnings. They distinguished among secondary, weak and strong embodiment. Proponents of secondary embodiment maintain that although embodiment exists, it is irrelevant for cognition *per se*. Rather, it is a consequence of inadvertent spreading activation across the cortical surface (Mahon and Caramazza, 2008; Chatterjee, 2010; Caramazza et al., 2014). Our analysis suggests that the spreading activation from the Associative Map to the F_2_ layer and from the F_2_ layer to the F_1_ layer is not an irrelevant byproduct of random cortical connectivity. Instead, it is a part of the neural architecture designed to achieve stability in learning and memory. We admit that the cognitive requirements of the task used in the study of Richter and Zwaan (2009) are minimal because participants just read the words. It may be argued that this analysis does not prove the necessity of perceptual simulation for conceptual understanding because the same behavioral effect is also consistent with the secondary embodiment. A more convincing argument would be to show how perceptual simulation helps during more-demanding tasks such as sentence comprehension (Stanfield and Zwaan, 2001; Zwaan et al., 2002). However, the modeling in such studies would require a more sophisticated neural network for sentence processing, which is beyond the scope of this paper. We argue that during sentence processing, the same pathway from the Associative Map to F_2_ and from F_2_ to F_1_ will be employed, and it will produce the same behavioral effect of slowing down the motor response in the mismatch condition.

At the other extreme, proponents of strong embodiment claim that conceptual processing occurs directly in sensory and motor systems (Gallese and Lakoff, 2005). Thus, there is no need for separate amodal conceptual representation. Furthermore, cognition and perception should be indistinguishable in functional imaging. However, the structure of the ART circuit, in which the F_0_ and F_1_ layers represent perceptual features (e.g., colors, shapes), whereas the F_2_ layer represents a more abstract grouping (categorization) of those features, suggests that the neural basis of concepts cannot be equated with those of perception. Consequently, perception and cognition will never activate exactly the same cortical surface. Cognition will always activate a region that is close but anterior to the area activated during perception. However, this anterior shift is not an argument against embodiment, as suggested by Rugg and Thompson-Schill (2013). Moreover, the inter-ART associative map has the property of the amodal conceptual representation, which connects to and binds together distinct modal representations in separate ART modules. The map's role in the conceptual processing is consistent with the activation of the anterior temporal lobe (Binder et al., 2009). In conclusion, it appears that weak embodiment, which combines properties of modal and amodal representations, is most consistent with the available behavioral and neural evidence (Meteyard et al., 2012; Pulvermüller, 2013) and with the computational mechanisms of the adaptive resonance theory.

## Conclusion

We showed how the real-time implementation of the ART neural network can successfully account for behavioral and functional neuroimaging data on grounded cognition (Martin, 2007; Barsalou, 2008). The interference effect found in behavioral tasks was attributed to the activation of the orienting subsystem. Anterior and overlap effects in fMRI studies were attributed to the layered structure of the attentional subsystem. Our simulations suggest that the behavioral and fMRI level of analysis are distinct manifestations of the same cortical circuit that is capable of solving a symbol grounding problem by perceptual simulation during conceptual processing as proposed by the theories of grounded cognition (Barsalou, 1999; Glenberg and Robertson, 1999; Zwaan, 2004). Moreover, perceptual simulation in the ART circuit arises naturally from the same neurocomputational mechanisms that are needed to achieve stability of learning and memory.

## Author contributions

All authors listed, have made substantial, direct and intellectual contribution to the work, and approved it for publication.

### Conflict of interest statement

The authors declare that the research was conducted in the absence of any commercial or financial relationships that could be construed as a potential conflict of interest.
