# Supplementary material for: Resonant Dynamics of Grounded Cognition: Explanation of Behavioral and Neuroimaging Data Using the ART Neural Network
Source: Front Psychol. 2016 Feb 11;7:139. doi: 10.3389/fpsyg.2016.00139 (PMC4749698; doi:10.3389/fpsyg.2016.00139)
Supplement: Supplementary file 1 [file Appendix.docx]

Appendix

The following mathematical description of the neural architecture for grounded cognition is divided into five sections. Section 1 describes the attentional subsystem with its three layers and two gain control units. Section 2 describes the orienting subsystem and its computation of activity match between the F_0_ and F_1_ layers. Section 3 describes how output from the F_2_ layer is transformed into motor response via leaky competitive accumulators (Usher & McClelland, 2001). Section 4 describes how averaged neural activity within model layers gives rise to a hemodynamic response (Arbib et al., 2000). Finally, section 5 discusses the choice of parameter values used in simulations and the model’s robustness to parameter changes.

The temporal evolution of neural activity or the membrane potential of the node $x_{p}^{\left(n\right)}$ in layer $n\in\left\{0,1,2,3\right\}$ at position $p\in\left\{i,j\right\}$ is described by a nonlinear shunting equation of the form

$\tau_{e}\frac{d}{dt}x_{p}^{\left(n\right)}=-x_{p}^{\left(n\right)}+\left(A-x_{p}^{\left(n\right)}\right)E_{p}^{\left(n\right)}-\left(B+x_{p}^{\left(n\right)}\right)I_{p}^{\left(n\right)}$.

In layers F_0_ and F_1_, the dimension of the network is *p = i = 1,…, M*. In layers F_2_ and F_3_, the dimension of the network is *p = j = 1, …, N*. In equation (1), $\tau_{e}$ represents an integration time constant; $-x_{p}^{\left(n\right)}$ represents the passive decay or leak toward the resting potential (which is assumed to be zero); A (B) represents the excitatory (inhibitory) saturation point, which constrains the node’s activity within the interval (-B, A); and $E_{p}^{\left(n\right)}$ $\left(I_{p}^{\left(n\right)}\right)$denotes total excitatory (inhibitory) input to the node (Grossberg, 1988). In all simulations, we used the same parameter set: $\tau_{e}$ = 10, *A* = 2, *B* = 2, *M* = 10, *N* = 4. The output or firing rate of the node $x_{p}^{\left(n\right)}$ is given by half-wave rectification

$f\left(a\right)=\left\{\begin{matrix} a & if & a>0 \\ 0 & if & a\leq0 \end{matrix}\right.$.

We adopted the convention of representing the supra-threshold activity of the node with capital letter *X*, that is,

$X_{p}^{\left(n\right)}=f\left(x_{p}^{\left(n\right)}\right)$.

Furthermore, we assumed that most of the excitatory input to the node arrives on the dendrites, which are modeled as independent computational units with their own nonlinear output function *h(a)*. Dendritic nonlinearity is modeled by a Heaviside step function

$h\left(a\right)=\left\{\begin{matrix} 1 & if & a>0 \\ 0 & if & a\leq0 \end{matrix}\right.$ .

There is plenty of evidence that dendrites are actively engaged in integrating synaptic inputs (reviewed in Häusser & Mel, 2003; London & Häusser, 2005). Based on these observations and computational modeling, Poirazi et al. (2003; Jadi et al., 2014) proposed a two-stage model of a single neuron where the output from the first or dendritic layer is summed at the second or somatic stage. The same approach is used in the current model because the dendritic output *h(a)* is summed at the soma and then subjected to the somatic output *f(a)*.

Furthermore, the same function *h(a)* is also used to model the computations in the gain control nodes and in the orienting subsystem and to model the output of comparison layers *C_1_* and *C_2_*. Inhibitory interneurons in the *F_0_*, *F_2_* and *F_3_* layers are not explicitly represented in the model. Instead, their instantaneous firing rates are inserted into the description of the dynamics of excitatory nodes. Differential equations are numerically solved using the LSODA routine on the interval from *t_0_* = 0 to *t_2_* = 100 subject to initial conditions $x_{p}^{\left(n\right)}\left(t_{0}\right)=0$ for all *n* and *p*.

A.1. Attentional Subsystem

A.1.1. F_0_ Layer

Excitatory input to the *i^th^* node of the *F_0_* layer is given by

$E_{i}^{\left(0\right)}=w_{e0}h\left[X_{i}^{\left(0\right)}+Z_{i}h\left(t\geq t_{1}\right)-0.2\right]$.

Excitatory input arrives on a dendrite that combines self-excitation $X_{i}^{\left(0\right)}$ with feedforward signal $Z_{i}$ arising from some earlier color-selective stage, and -0.2 denotes the dendritic threshold. The dendritic impact on the node is modulated by the parameter *w_e0_*, which could be interpreted as a dendritic weight. The color code shown in Figure 3A implies that the presentation of pure red color produces the following input pattern:

$Z=\left[\begin{matrix} 1 & 1 & 1 & 1 & 0 & 0 & 0 & 0 & 0 & 0 \end{matrix}\right]$.

The input is switched on at *t_1_* = 40. The inhibitory input to the *i^th^* node in *F_0_* is given by

$I_{i}^{\left(0\right)}=w_{s0}\sum_{k\neq i} f\left(X_{k}^{\left(0\right)}-X_{i}^{\left(0\right)}-0.05\right)$.

Equation (7) describes lateral inhibition arriving from the *k^th^* node within the *F_0_* layer. The strength of the lateral inhibition is controlled by the parameter *w_s0_*. Lateral inhibition is modulated by the recurrent self-inhibition, which acts upon the axons of the *k^th^* node. The biophysical interpretation of this computational design is that the soma of the *i^th^* node is able to release a neurotransmitter that binds to the axonal endings of the *k^th^* node if they are endowed with appropriate receptors. Neurophysiological studies suggest that many neurotransmitters, including endocannabinoids, GABA and glutamate, are involved in this type of retrograde signaling throughout the brain (Alger, 2002; Regehr et al., 2009; Zilberter, 2000; Zilberter et al., 2005). From the computational perspective, this is a form of presynaptic inhibition, which has been used previously in the modeling of lateral interactions in the cortex (Yuille & Grzywacz, 1989; Spratling & Johnson, 2002; Domijan & Šetić, 2007). The consequence of pre-synaptic inhibition is that the *F_o_* layer will be able to exhibit object-based winner-takes-all behavior, that is, it will be able to suppress background activity, but it will allow multiple nodes to simultaneously remain active if they encode the same feature. The parameters of the *F_0_* layer were set to *w_e0_* = 2, *w_s0_* = 4.

A.1.2. F_1_ Layer

Excitatory inputs to the *i^th^* node in the *F_1_* layer arrive from two independent dendrites

$E_{i}^{\left(1\right)}=h\left(X_{i}^{\left(0\right)}-0.2\right)+h\left(\sum_{j} X_{j}^{\left(2\right)}w_{ji}-0.2\right)$.

The first dendrite receives feedforward input from the *F_0_* layer, and -0.2 denotes the dendritic threshold. The second dendrite receives feedback or top-down projections from the *F_2_* layer. A matrix of synaptic weights *w_ji_* that modulates the signal strength in the top-down pathway from the *j^th^* node in the *F_2_* layer to the *i^th^* node in the *F_1_* layer is given by:

$w_{ji}=\left[\begin{matrix} 1 & 1 & 1 & 1 & 0 & 0 & 0 & 0 & 0 & 0 \\ 0 & 1 & 1 & 1 & 1 & 0 & 0 & 0 & 0 & 0 \\ 0 & 0 & 0 & 0 & 0 & 1 & 1 & 1 & 1 & 0 \\ 0 & 0 & 0 & 0 & 0 & 0 & 1 & 1 & 1 & 1 \end{matrix}\right]$.

We assume that all synaptic weights in the model are fixed and learned prior to the start of simulation. Inhibitory input to the *i^th^* node in the *F_1_* layer arises from the gain control node *g_1_*:

$I_{i}^{\left(1\right)}=f\left(g_{1}\right)$.

The temporal evolution of the activity of the gain control node *g_1_* is described by the additive equation of the form

$$\tau_{i}\frac{d}{dt}g_{1}=-g_{1}+h\left(\sum_{j} X_{j}^{\left(2\right)}-0.2\right)+P_{1}$$

Parameter $\tau_{i}$ is time constant for the inhibitory node, which is assumed to be faster relative to excitatory nodes. The gain control node receives excitation from the node that computes the total activity arising from *F_2_*. Parameter *P_1_* controls whether the *F_1_* layer is actively engaged in perception or is in a passive state. In the simulation of behavioral data, it was always set to *P_1_* = 0, indicating active engagement of *F_1_*. In the simulation of fMRI data, it was set to *P_2_* = 2 in the condition of passive perception and again to *P_2_* = 0 during active perception. The time constant for the gain control node was set to $\tau_{i}=1$.

A.1.3. F_2_ Layer

Excitatory input to the *j^th^* node in the *F_2_* layer is defined by

$$E_{j}^{\left(2\right)}=\sum_{i} X_{i}^{\left(1\right)}w_{ij}+w_{e2}h\left(X_{j}^{\left(2\right)}+\sum_{q} X_{q}^{\left(4\right)}w_{qj}-0.2\right)$$

The first term on the right-hand side of equation (12) describes an adaptive filter that computes a dot product between a vector of neural activity registered at preceding layer *F_1_* and a vector of long-term memory traces or synaptic weights. A matrix of synaptic weights *w_ij_* that modulates the signal strength in the bottom-up pathway from the *i^th^* node in the *F_1_* layer to the *j^th^* node in the *F_2_* layer is given by the transpose of the matrix of top-down weights defined by equation (9):

$w_{ij}=w_{ji}$.

Separately from the somatic input, the dendrite of the *j^th^* node receives self-excitation and feedback signals from the associative map $X_{q}^{\left(4\right)}$, which enables perceptual simulation to occur. For simplicity, we considered just two nodes in the associative map that correspond to the representation of the concepts RED and GREEN. When the concept RED is activated, we set $X_{1}^{\left(4\right)}=1$ and $X_{2}^{\left(4\right)}=0$. The opposite setting is used for concept GREEN. When there is no perceptual simulation, both nodes are set to 0. A matrix of synaptic weights from the associative map to *F_2_* is given by

$w_{qj}=\left[\begin{matrix} 1 & 1 & 0 & 0 \\ 0 & 0 & 1 & 1 \end{matrix}\right]$.

The inhibitory input to the *j^th^* node in the *F_2_* layer consists of three sources:

$$I_{j}^{\left(2\right)}=f\left(g_{2}\right)+w_{s2}\sum_{k\neq j} f\left(X_{k}^{\left(2\right)}-X_{j}^{\left(2\right)}-0.05\right)+w_{r}f\left(x_{j}^{\left(3\right)}-0.2\right)$$

The first source of inhibition is the gain control node *g_2_*, which inhibits the *F_2_* layer while *F_0_* is silent. The second source of inhibition in equation (15) is lateral inhibition among *F_2_* nodes. It has the same form of recurrent pre-synaptic inhibition as described for the *F_0_* layer. In this case, presynaptic inhibition ensures that the *F_o_* layer will be able to exhibit winner-takes-all behavior during perception and categorization, but at the same time it will enable multiple nodes to simultaneously remain active during perceptual simulation. The third source of inhibition in equation (15) is the activity from the orienting subsystem described in Section 2.

The temporal evolution of the activity of the gain control node *g_2_* is described by the additive equation of the form

$\tau_{i}\frac{d}{dt}g_{2}=-g_{2}+h\left(\sum_{j} X_{j}^{\left(2\right)}-0.2\right)+P_{2}-f\left[h\left(\sum_{i} X_{i}^{\left(0\right)}-0.2\right)-P_{1}\right]$.

It receives excitation from the node that computes the total activity of *F_2_* and inhibition from the node that computes the total activity of *F_0_*. In this way, *F_2_* inhibits itself during perceptual simulation. However, the registration of input at *F_0_* disinhibits *F_2_*. Parameter *P_2_* controls whether *F_2_* is actively engaged in cognitive processing or is in a passive state. In the simulation of behavioral data, it is always set to *P_2_* = 0, indicating active engagement of *F_2_*. In the simulation of fMRI data, it is set to *P_2_* = 2 in the passive condition and again to *P_2_* = 0 in the active condition. Disinhibition of gain control unit *g_2_* by the activity of the F_0_ layer is prevented by *P_1_* in the state of passive perception. In this way, the F_0_ layer cannot generate output to the *g_2_* unit and interfere with active conceptual processing. The other parameters of the *F_2_* layer were set to: *w_e2_* = 2, *w_s2_* = 4, *w_r_* = 4.

A.2. Orienting Subsystem

Excitatory input to the *j^th^* node in the *F_3_* layer is given by

$E_{j}^{\left(3\right)}=w_{e3}h\left[X_{j}^{\left(3\right)}-0.2\right]+h\left[X_{j}^{\left(2\right)}+f\left(r\right)-2\right]$.

It receives self-excitation on one dendrite, and the impact of self-excitation on the node is modulated by *w_e3_*. Additionally, it receives input from *F_2_* on a separate dendrite together with the input from the *R* node, which computes the degree of match between the total activity of the *F_0_* and *F_1_* layers. The second dendrite has a high threshold so that it computes logical AND between the inputs from the *F_2_* and the *R* node.

The temporal evolution of the neural activity of the *R* node is described by the additive equation of the form

$\tau_{r}\frac{d}{dt}r=-r+h\left[\rho\sum_{i} h\left(X_{i}^{\left(0\right)}-0.2\right)-\sum_{i} h\left(X_{i}^{\left(1\right)}-0.2\right)\right]+h\left[\sum_{j} h\left(X_{j}^{\left(2\right)}-1\right)\right]-1$.

Parameter $\tau_{r}$ is time constant for the *R* node, which is assumed to be faster relative to excitatory nodes. The second term on the right hand side of equation (18) describes a comparison between total activity arriving from *F_0_* and *F_1_*. It will generate a positive (or mismatch) signal to the *R* node when

$\frac{\sum_{i} h\left(X_{i}^{\left(1\right)}-0.2\right)}{\sum_{i} h\left(X_{i}^{\left(0\right)}-0.2\right)}<\rho$.

To prevent undesirable activation of the *R* node during the first pass through the $F_{0}\to F_{1}\to F_{2}$ pathway, we introduced a recurrent projection from *F_2_* to the *R* node. In this way, the reset signal can only be triggered if the *F_2_* layer is fully activated. The last term in equation (18) describes tonic inhibition. The parameters of the orienting subsystem were set to: $\rho$ = 0.9, *w_e3_* = 2, and $\tau_{r}$ = 5. For the simulation shown in Figure 6, the time constant for the *R* node was set to $\tau_{r}$ = 1 in Figure 6A; to $\tau_{r}$ = 5 in Figure 6B; and to $\tau_{r}$ = 10 in Figure 6C.

A.3. Simulation of Behavioral Response

We modeled decisions about correct responses using a leaky competitive accumulator model without noise (Usher & McClelland, 2001). Evidence accumulation for the Same unit is described by the additive equation of the form

$\tau_{m}\frac{d}{dt}x^{\left(S\right)}=J_{0}+h\left[\sum_{j} h_{c1}\left(X_{j}^{\left(2\right)}+X_{j}^{\left(5\right)}-2.5\right)-0.5\right]-\alpha x^{\left(S\right)}-\beta X^{\left(D\right)}$.

In equation (20), $\tau_{m}$ is the time constant for evidence accumulation and *J_0_* is non-specific excitatory input. The second term describes the output of the first comparison layer *C_1_*, which computes logical AND between nodes at the *j^th^* position in *F_2_* and *F_5_*. The dynamics of the F_5_ layer are not explicitly represented. Instead, its steady state activity is set to $X_{j}^{\left(5\right)}=1.5$ if the *j^th^* F_2_ node was activated during the perception of the reference stimulus or to $X_{j}^{\left(5\right)}=0$ otherwise. Activity in the F_5_ layer was set to 1.5 because it is close to the level of steady state activity in the F_2_ layer, which enables direct comparison of activity between two layers. Parameter $\alpha$ represents the rate of passive decay or leak, and $\beta$ represents the strength of lateral inhibition between the Same and Different unit. In the same manner, evidence accumulation for the Different unit is given by

$\tau_{m}\frac{d}{dt}x^{\left(D\right)}=J_{0}+h\left[\sum_{j} h_{c2}\left(X_{j}^{\left(2\right)}+X_{j}^{\left(5\right)}-0.5\right)-1.5\right]-\alpha x^{\left(D\right)}-\beta X^{\left(S\right)}$.

The Different unit receives input from the second comparison layer *C_2_*, which computes logical OR between nodes at the *j^th^* position in *F_2_* and *F_5_*. The Different unit will become active only if at least two distinct *C_2_* nodes are simultaneously active.

To translate simulated time steps into an observable response, we applied following transformation

$$\psi^{\left(S/D\right)}=Q+\min\left\{T:X^{\left(S/D\right)}\left(T\right)>0.5\right\}\Delta t$$

where Q represents total non-decision time, that is, the time necessary to complete sensory encoding and motor execution. The second term in equation (22) represents decision time, that is, the time needed to cross the threshold of 0.5 and to initiate the response. The time $T=f\left(t-t_{1}\right)$ is shifted relative to the start of the input presentation at *t_1_*. Term $\Delta t$ translates arbitrary simulation units into milliseconds. The parameters were set to: $\tau_{m}$ = 10, *J_0_* = 0.2, $\alpha$ = 1, $\beta$ = 0.1, *Q* = 700 ms, $\Delta t$ = 3 ms.

A.4. Simulation of Hemodynamic Response

We assumed that the metabolic demand that drives regional cerebral blood flow , generated by the model layer *n*, is approximately proportional to the space-time average $\overline{X}_{p}^{\left(n\right)}$ of its total supra-threshold activity computed over the temporal interval from *t_0_* = 0 to *t_2_* = 100:

.

Alternatively, the space-time average $\overline{X}_{p}^{\left(n\right)}$ could be defined as an absolute value of the total membrane potentials to better reflect excitatory and inhibitory post-synaptic potentials, as in

.

In equations (23) and (24), *K_n_* is a scaling parameter that depends on the size of the layer and the total duration of observation. In the simulations, it was set to: *K_0_* = 2000, *K_1_* = 2200, *K_2_* = 1000.

A.5. Parameters

The model contains many free parameters, some of which are critical for the model’s success. Here, we identify those parameters and how we set them. The crucial parameters for the simulation of behavioral data were integration time constants for the excitatory nodes $\tau_{e}$, gain control units $\tau_{i}$, and the R node $\tau_{r}$. We set $\tau_{e}$ and $\tau_{i}$ to arbitrary values and then we systematically varied $\tau_{r}$ in order to observe its influence on the model’s dynamics.

As shown in Figure 6, when the dynamics of the R node are slowed down, by increasing $\tau_{r}$from 1 to 10, the result was an increase in the magnitude of interference in the mismatch condition without any effect on the facilitation in the match condition. The observed facilitation effect is weak due to the nonlinear shunting model of a neuron described by equation (1). In particular, the nonlinear term $\left(A-x_{p}^{\left(n\right)}\right)$ in equation (1) forces the membrane potential to quickly reach its maximal level, thus destroying any temporal advantage produced by the match condition. Furthermore, we observed that the variation of time constant of the gain control units $\tau_{i}$ in the range 1 to 10 did not alter the model’s behavior.

Crucial parameters for the simulation of imaging data were *P_1_* and *P_2_*, describing external inhibition, which controls whether the network is in an active or a passive state. They were set to 2 in the passive state to completely inhibit the target layer and any larger value would have the same effect. Furthermore, dendritic thresholds could be altered by 50% without a noticeable effect on the model’s output. Exceptions to this rule are the dendritic threshold for nodes in the F_3_ layer, which showed a 10% tolerance to change, and the dendritic threshold in the pathway from the F_2_ layer to the R node, which showed a 30% tolerance to change. The threshold for presynaptic inhibition was chosen to be small (0.05) because it controls the precision of the competition in the F_0_ and F_2_ layers. Larger values of this parameter would destroy their ability to exhibit winner-takes-all behavior. Finally, synaptic weights in the inhibitory pathway from the F_3_ to F_2_ layers, which delivers the reset signal, were chosen in a way to achieve the desired effect of inhibiting the currently active F_2_ node. Weaker values of this synaptic weight would destroy the network ability to reset the F_2_ layer.
